# Recent advancements in the analysis of bone microstructure: New dimensions in forensic anthropology

**DOI:** 10.1080/20961790.2018.1483294

**Published:** 2018-10-03

**Authors:** Janna M. Andronowski, Christian Crowder, Miriam Soto Martinez

**Affiliations:** a Department of Biology, The University of Akron, Akron, OH, USA;; b Harris County Institute of Forensic Sciences, Houston, TX, USA

**Keywords:** Forensic science, forensic anthropology, cortical bone, synchrotron, micro-CT, *in vivo*, *ex vivo*, histological age estimation, bone quality

## Abstract

Bone is a mechanically active, three-dimensionally (3D) complex, and dynamic tissue that changes in structure over the human lifespan. Bone tissue exists and remodels in 3D and changes over time, introducing a fourth dimension. The products of the remodelling process, secondary and fragmentary osteons, have been studied substantially using traditional two-dimensional (2D) techniques. As a result, much has been learned regarding the biological information encrypted in the histomorphology of bone, yielding a wealth of information relating to skeletal structure and function. Three-dimensional imaging modalities, however, hold the potential to provide a much more comprehensive understanding of bone microarchitecture. The visualization and analysis of bone using high-resolution 3D imaging will improve current understandings of bone biology and have numerous applications in both biological anthropology and biomedicine. Through recent technological advancements, we can hone current anthropological applications of the analysis of bone microstructure and accelerate research into the third and fourth dimensional realms. This review will explore the methodological approaches used historically by anthropologists to assess cortical bone microstructure, spanning from histology to current *ex vivo* imaging modalities, discuss the growing capabilities of *in vivo* imaging, and conclude with an introduction of novel non-histological modalities for investigating bone quality.

## Introduction

Histological analysis of bone has a long history in the relatively young field of forensic anthropology. Early research by Kerley [1], Jowsey [2], Ahlqvist and Damsten [3], and Singh and Gunberg [4], for example, explored qualitative and quantitative differences in human and non human cortical bone. Histological research within forensic anthropology is typically focused on the analytical outcome, meaning the ability to accurately estimate age at death or differentiate human from the non human bone. Thus, research often focuses on method improvement, which is understandable considering the applied nature of the field. While this continues to remain an important goal of bone histology research, efforts over the past decade have directed greater attention to understanding bone biology using a variety of technological developments to study bone in new ways. Prior to discussing these technological advances, it is important to review certain aspects of bone biology and important histological research within forensic anthropology.

**Figure 1.**
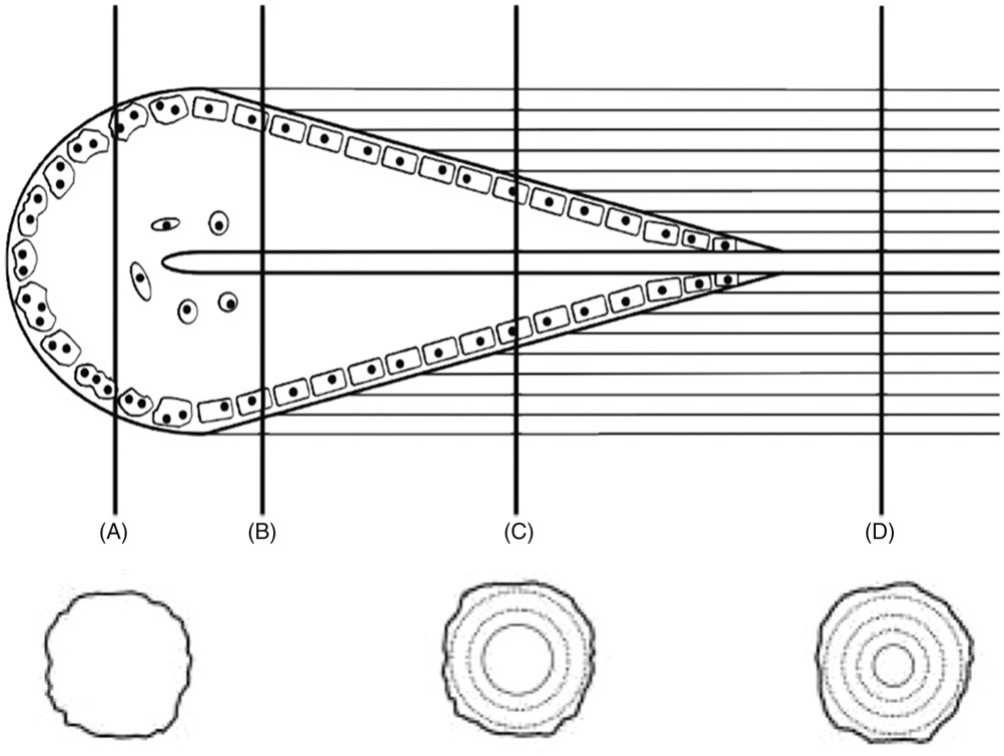
Coupled osteoclast and osteoblast activity depicted as a Basic Multicellular Unit (BMU) during bone remodelling. (A) Osteoclastic activity near the leading edge of the cutting cone, (B) initiation of osteoblastic activity, (C) active osteoblast activity, and (D) a fully formed intact osteon depicting a Haversian canal in the centre. Adapted from Pratt, with permission.

Bone’s remarkable ability to repair microdamage, respond to load-bearing phenomena, and perform homeostatic functions is accomplished through remodelling. Remodelling is a tissue turnover process that occurs without changing the macroscopic bone architecture. The remodelling process is continuous throughout life and involves a complex arrangement of cells called Basic Multicellular Units (BMUs). First described by Frost [5], BMUs remodel bone via the removal and replacement of “packets” of bone called bone structural units (BSUs). Remodelling follows an activation, resorption, and formation (ARF) sequence. The series can be further expanded to six phases that includes activation, resorption, reversal, formation, mineralization, and quiescence [6]. Remodelling removes and replaces older and/or damaged bone, allowing the bone to maintain its mechanical competence. BMUs move through tissue space by tunnelling through the bone material (Figure 1) with a leading region lined with osteoclasts. The diameter of the excavation tunnel is determined by osteoclast activity and corresponds with the size of newly formed osteons. As BMUs transition between resorption and formation, a cement or “reversal line” is formed. Osteoblasts attach to the reversal lines, and line the resorption tunnel. Osteoblasts deposit osteoid in a concentric pattern from the outer edge of the tunnel inwards, leaving a Haversian canal in the centre of the newly formed osteon.

Bone remodelling has three purposes: (1) alter the balance of essential nutrients in the body by increasing and/or decreasing serum concentration [7], (2) protect bone from natural biomechanical forces that cause microscopic damage, and (3) repair microdamage to prevent the development of macrodamage. Maintaining bone homeostasis involves complex interactions at the molecular, cellular, and tissue level. Osteocytes play a pivotal role in mechanotransduction and transmission of information to the effector cells (osteoclasts and osteoblasts). It is essential to recognize these interactions as a cohesive operating system that allows for the maintenance of the gross morphology, microarchitecture, and mechanical properties of bone. The on going remodelling of bone over the lifespan produces indicators of skeletal health and provides information regarding skeletal age.

Remodelling has long been viewed as multifaceted, influenced by physiological and mechanical functions [8]. In recent years, efforts to explore the multi-functional roles of the remodelling process have been aided by three-dimensional (3D) modalities used to explore the products produced by remodelling. This review will survey a range of approaches used for the visualization and analysis of bone tissue spanning from traditional histology to current *ex vivo* 3D imaging modalities, cutting-edge *in vivo* four-dimensional (4D) imaging techniques, and concluding with the introduction of novel non-histological modalities for investigating bone quality.

## Traditional two-dimensional (2D) perspectives

### Histological age estimation

Bone was among the first biological tissues to be studied microscopically and traditional light microscopy continues to yield a wealth of information about bone microstructure. Early applications revealed structures related to remodelling including vascular canals, resorption spaces and mature osteons. The relationship between bone remodelling and age was documented among very early observations of ground bone sections [9]. The resulting quantification of bone microstructural features related to age-at-death estimation has been widely used by skeletal biologists.

Histological age estimation is grounded in the study of BSUs. BSUs consist of secondary osteons that undergo remodelling over the lifespan and their relative density increases with age [10]. Both intact and fragmentary osteons comprise an osteon population density (OPD) variable used for histological age estimation. With increasing age, the cortical bone becomes crowded with secondary osteons until an asymptote is reached in osteon counts [10]. Therefore, as evidence of previous secondary osteons are erased, the OPD value plateaus or slightly decreases. Two factors, osteon size and cortical thickness, are thought to contribute to the age at which OPD will reach an asymptote. As such, this renders histological age estimation methods especially imprecise and biased when applied to older individuals. Regardless, various histological age estimation methods have been developed using human cortical bone tissue [1, 11, 12]. These methods use specific skeletal elements, with the majority of research focusing on the rib and femur.

#### Microscopic methods: rib

Histological methods for the rib [12, 13] are largely based on clinical research performed by Frost et al. [5, 14–18] at the Henry Ford Hospital during the 1960s and 1970s. Influenced by this research, Stout [13] developed the first histological age estimation method using the mid-third of the sixth rib. Intact and fragmentary osteon counts were combined to produce the OPD variable. Using the rib as a sampling location attempted to bypass the influence of biomechanical factors on weight-bearing bones. For an in-depth discussion regarding histological age-estimation methods using the rib, the following references are suggested [12, 13, 32].

#### Microscopic methods: femur

The subperiosteal cortex of the mid-shaft femur is another traditionally employed sampling location [1, 3, 11, 19, 20]. Kerley [1] developed an early histological age estimation method using transverse cross-sections from the femur, tibia, and fibula. The Kerley [1] method claimed simplicity and repeatability, yet in retrospect the study foreshadowed difficulties to come in using osteon counts from a specific field size. Variations of the Kerley method or different approaches based on the premise of Kerley’s work have provided a wealth of publications over the years [3, 4, 11, 19, 21–25]. Since the publication in 1965, discussions regarding sex-specific equations, sampling location, and variable definitions have been debated among researchers. Despite these arguments, there are two primary commonalities that occur: (1) the use of a linear model to estimate age at death centred on the assumption that the replacement of primary bone with secondary bone is a continuous process of turnover that occurs at a predictable rate [23, 25], and (2) the number of intact and fragmentary secondary osteons per unit of cortical area is considered to be the best indicator of skeletal age at death. Future research will likely challenge the use of linear models and a deeper understanding of bone biology will drive this endeavour. Investigating the influences of extrinsic and intrinsic considerations such as biomechanical stressors, taphonomy, disease, trauma, substance abuse, diet and nutrition, hormones (e.g. vitamin D, estrogen), and asymptotic values [10, 23, 26] provide avenues for future research as their influences are not fully understood. For an in-depth review of histological age estimation methods developed or influenced by Kerley’s [1] original publication, the following references are recommended [3, 4, 11, 19, 21, 22, 24, 25].

### Determining human from non human bone

In contexts where skeletal remains are frequently highly fragmented, degraded, burnt, or otherwise unidentifiable, it has proven useful to apply traditional 2D histological approaches to infer species origin. The qualitative analysis of cortical bone primarily relies upon the identification of bone tissue type, pattern, and organization. For example, mammalian bone may contain fibrolamellar, lamellar, woven, and/or Haversian bone tissue types [27]. Laminar fibrolamellar bone may be distinguished by circumferential lamellar layers. A brick-like pattern of fibrolamellar bone, often referred to as plexiform bone, is commonly found in non human mammalian bone and is distinguished by alternating sheets of woven and lamellar bone [27, 28]. This plexiform-type arrangement of fibrolamellar bone is rarely observed in adult human bone.

Secondary osteonal bone, or Haversian bone, is more difficult to distinguish between human and non human species. This bone type refers to bundles of lamellar bone tissue with a central Haversian (vascular) canal. The concentric lamellae are defined by a reversal line, which is the characterizing feature of secondary osteonal bone tissue. Haversian bone is documented in various reptilian, avian, and mammalian species, including humans [29]. Thus, the presence of Haversian bone alone is not diagnostic of human bone. Methods to ascertain human from non human bone that relies on differences in Haversian canal size, and osteon size/shape have been proposed, but employed with varying degrees of success. Thus, use of these methods should be undertaken with caution as most remain in need of validation [30]. The following key publications are recommended for a detailed discussion regarding the biology of human and non human bone [2, 27, 31, 32].

#### Limitations of current 2D histologic methods

Various methodological issues exist regarding the current application of histological age estimation methods. One such issue involves the difficulty in identifying intact and fragmented osteons, considering that definitions of these features vary between researchers. Definitions are often ambiguous or require subjective classifications by the observer, resulting in high inter-observer error and/or problems making cross-study comparisons. Morris and Crowder [33] reported the level of observer error associated with variable definitions relating to OPD as defined by Stout and Paine [12] and Cho et al. [34]. Results indicated that separating out intact osteon population density (iOPD) and fragmentary osteon population density (fOPD) increased error and that the OPD variable should be used considering that misclassified osteons would likely be captured when adding the two variables. In other words, missed intact osteons would be captured in the fragmentary counts and since the method regression equation only factors in OPD, the error would be negated. In response to the 2005 study, Heinrich et al. [35] proposed new definitions for intact and fragmented osteons that were designed to limit observer subjectivity and focus on the biological significance of each osteon group. Observations of iOPD and fOPD were made by three observers and these observations were used to explore the inter-observer error and biological significance associated with the proposed definitions. Results indicate that the proposed definitions significantly reduce inter-observer error and misidentification of intact and fragmented osteons. It was noted that the inter-observer error associated with fragmented osteons was still high. The age-related biological significance examined using these definitions demonstrated that age-related accumulation of intact and fragmented osteons is not equivalent. While the literature suggests combining iOPD with fOPD to reduce observer error, the results suggest that doing so may reduce the ability to interpret bone remodelling. The authors suggest capturing the variable separately, using the proposed definitions, and re-developing the regression equations for future studies.

While the nomenclature issues discussed above are not isolated to 2D analyses, it should be noted that they are partially a product of 2D techniques. Overall, a common limitation of traditional histological modalities is that they are 2D in nature, thus providing a limited window into the inner workings of bone. Researchers continue to benefit greatly from 2D methodologies as they often provide better resolution for microstructural details and cellular characteristics than other approaches, especially when sophisticated staining techniques are applied. Recent 3D imaging modalities are accelerating research in the field of bone biology [36, 37] and provide a more comprehensive understanding of cortical microstructure, thus offering new tools for biological/forensic anthropologists.

## Historical perspectives: a brief history of three-dimensional (3D) imaging

In the mid-late 1600s, Anthony Van Leeuwenhoeck published what is thought to be the first description of cortical bone histology and expressed appreciation of its 3D structure [38]. He described the arrangement of cortical bone tissue as a series of “pipes” and penned a 3D render of his observations. Clopton Havers further described types of pores that run transversely and longitudinally through cortical bone tissue to its surface. These early descriptions were based on observations of blocks of bone using low magnification.

Past 3D investigations of bone microstructure were based on time consuming and tedious protocols which employed two approaches: (1) staining and/or casting of cortical canals, and (2) serial section reconstruction. Casting and/or staining bone specimens [39] allowed researchers to determine the positioning of vascular canals, the spatial orientation of cortical bone, and osteon orientation [40, 41]. Hert et al. [40], for example, filled the vascular canals of undecalcified cortical bone with India-ink to assess the spatial microarchitecture. Other projects employing casting/staining techniques examined canal networks in various animal species, and evaluated resorptive bay distribution [42, 43]. Though allowing for visualization of canals, these techniques did not provide enough quantitative data to visualize the entire 3D structure of bone samples.

As demonstrated by Cohen and Harris [44], serial sectioning and visualization using light microscopy allowed for the examination of vascular canals in cortical bone. Sterio [45] and Gunderson [46] established that the number of cells per volume could be measured through serial sectioning using what is called the “dissector method”. Additionally, Tappen [47] visualized resorptive bays evident during the remodelling process in canine bone. Reconstructed 3D sections also provided researchers with the necessary information to create 3D models out of various materials such as wire and paper [9, 44]. More recently, advancing computer technology has allowed for much more efficient 3D rendering and automation of serial sections. Stout et al. [48], for example, used serial section reconstruction to analyze the 3D nature of osteons in canine bone. The authors discovered that osteons appeared to have complicated and interconnecting branching patterns, thus refuting earlier evidence suggesting that osteons display spiralling organization. A notable finding by Stout et al. [48] revealed that previously described “dumb-bell shaped” osteons are in fact bi-products of the 2D plane of sectioning. Although providing 3D information, serial sectioning and subsequent reconstruction require destruction of study specimens, produce generally qualitative results, and are tedious in nature.

Hounsfield developed the first commercial quantitative computed tomography (QCT) system in 1973. Prior to the development of QCT, a screening and diagnostic tool referred to as digital tomosynthesis allowed for the clinical identification of pathological abnormalities in soft tissue structures (e.g. breast tumours). This early imaging modality was limited in its access to 3D information and out of plane structures were often illuminated subsequently blurring the target object. Hounsfield’s traditional CT system relied on differential X-ray absorption across materials or tissues. A series of projection X-ray images could be taken of a rotated object from 0 to 180 degrees. From these projections, the 3D structure of the object can be reconstructed. In reconstructed images, the grey levels are inverted resulting in the denser materials being darker and areas of high absorption being brighter.

Boushey et al. [49] published an early study employing QCT in a clinical context to evaluate osteoporosis. The authors employed QCT in the examination of lumbar vertebral mineral content. This technique afforded high precision compared to alternative imaging modalities available at the time of publication including radiogrammetry and photon absorptiometry. QCT has also been employed to measure volumetric bone mineral density (BMD), rather than 2D areal BMD, and can be applied to measure the geometric properties of long bones [50–52].

Findings from these conventional 3D imaging techniques demonstrated the need for further volumetric analysis of cortical bone microarchitecture. As a result, early 3D approaches have been superseded by the use of micro-Computed Tomography (micro-CT) for the study of bone structure and quality.

## Current perspectives: *ex vivo* 3D imaging

### Micro-Computed Tomography

Micro-CT has been regularly used since 1989 as an approach for analyzing the 3D microstructure of cancellous bone [53]. The original application of micro-CT to bone microstructure was to assess and quantify pathological changes and mechanical properties of bone associated with osteoporosis *in vitro* [54, 55]. The ability to quantify 3D bone architecture efficiently, and with software developed for cancellous bone analysis, is a very important advantage associated with the micro-CT approach. Micro-CT also offers the benefit of preservation of the bone specimen, as opposed to former more destructive techniques such as serial sectioning. There are two primary micro-CT systems that can be applied to the examination of bone tissue: (1) laboratory or desktop micro-CT, and (2) synchrotron radiation-based micro-CT (SR micro-CT).

#### Micro-CT: desktop

Laboratory or desktop micro-CT systems have become the “gold standard” for the non-destructive 3D analysis of cancellous bone since its introduction by Feldkamp et al. [53]. These self-contained commercial systems use polychromatic microfocus X-ray tubes to image specimens at high resolutions. The potential of micro-CT to fields such as bone biology and anthropology was quickly recognized. The desktop systems have been used broadly and studies span from the evaluation of cancellous bone morphology in humans and non human primates [56–58], paleopathological analysis [59], to the analysis of teeth and examination of fossil remains [60]. The resolution of these systems is limited by the spot size and energy of the X-ray tube, which is mutually exclusive. The non-destructive nature of micro-CT and its capacity for the 3D visualization and analysis of microstructural features, however, are key factors continuing to drive its use in both bone biology and anthropology. The capabilities of micro-CT also minimize or eliminate inconsistencies with sample site location due to the ability to image larger volumes of bone, or entire bones. Microstructural parameters can further be measured volumetrically, and the interpolation of 3D data from 2D sections is no longer necessary [61].

The application of micro-CT to cortical bone remains challenging as the internal structures are much smaller than trabeculae. The consistent visualization of cortical bone porosity, for example, requires resolutions of ten microns or higher [62]. Measurements such as volumetric BMD, bone volume fraction (BV/TV), intra-cortical porosity, and specific surface of the bone (BS/TV), however, are obtainable for cortical bone. The bone volume fraction is the percent of bone tissue within a given Volume of Interest (VOI). The bone volume fraction is an important determinant of load bearing capacity [63, 64]. Intra-cortical porosity is a measure of the porosity within cortical bone and is specifically measured as the amount of the VOI not occupied by bone tissue. Increased intra-cortical porosity is associated with decreased fracture toughness [65]. Bone-specific volume measures the amount of bone surface area to VOI. Greater bone-specific volume is associated with increased intra-cortical porosity [66]. Bone volume fraction can also be obtained from cancellous bone, as well as trabecular thickness, separation, and connectivity [67]. At the millimetre level, these measurements are associated with tissue level (cortical and cancellous) mechanical properties. Predominantly, the use of micro-CT is restricted to human bone samples obtained postmortem or via bone biopsy, although *in vivo* micro-CT scanners have been recently developed and applied to studies of bone structure and bone health [61, 68, 69].

The visualization of histological features beyond vascular canals, however, remains out of reach. The use of more sophisticated X-ray sources, such as synchrotron facilities, is narrowing the gap between traditional histology and 3D imaging for bone tissue.

#### Micro-CT: synchrotron radiation micro-CT

In SR micro-CT facilities, X-rays are produced by the passage of an electron beam that travels close to the speed of light through electromagnets. The generated X-ray beam travels down a beamline where it can be optionally filtered by a monochromator. As a result, radiation of a specific wavelength or energy can be selected. The flux, or number of X-ray photons passing through an area over a given time, is much greater in magnitude than desktop micro-CT systems, and high-resolution submicron level imaging can be achieved. The brilliance of radiation can produce high-resolution images of spectacular quality, reduced scan times, and faster and more accurate quantitative measurements [60, 70, 71].

SR micro-CT is revolutionizing the current understanding of bone structural biology by contributing novel 3D data. SR micro-CT has recently been extended to examining human bone at the cellular level, which will continue to contribute to the understanding of bone adaptation, disease, and aging [60, 70–74]. With resolutions of 1–2 µm, SR micro-CT technology has further allowed researchers to quantitatively analyze osteocyte lacunae. Since osteocytes are soft tissue structures which are deeply encased within the bone matrix, they cannot be visualized using available X-ray imaging techniques. As such, their associated cellular spaces (lacunae) are used as substitutes. Although former studies have examined osteocytes and their lacunae in both human and nonhuman animals [75–78], quantifying osteocyte population density has been problematic due to limitations of traditional lower-resolution 2D imaging techniques [71, 79].

SR micro-CT imaging, however, has recently allowed for hundreds of thousands of osteocytes to be visualized and analyzed [70–72, 74]. This application has presented an opportunity to consider osteocytes individually, as well as examine them in terms of the larger population. Carter et al. [70] were the first to examine osteocyte lacunar density and morphology between cross-sectional regions in human femora, and evaluated osteocyte morphology, spatial distribution, regional variation, and density. Results indicated extensive regional variation (about 30%) in osteocyte lacunar density among anatomical segments [71]. In terms of lacunar shape, the anterior and posterior regions revealed more elongated lacunae than other regions. Most notably, the authors reported a higher total number of osteocyte lacunae compared to previous studies based on 2D imaging techniques. These differences may be attributed to the employment of SR micro-CT and the ability to evaluate a larger VOI in 3D. More recently, the application of osteocyte lacunar parameters has been applied to inquiries in forensic anthropology [72].

## New dimensions in forensic anthropology

**Figure 2.**
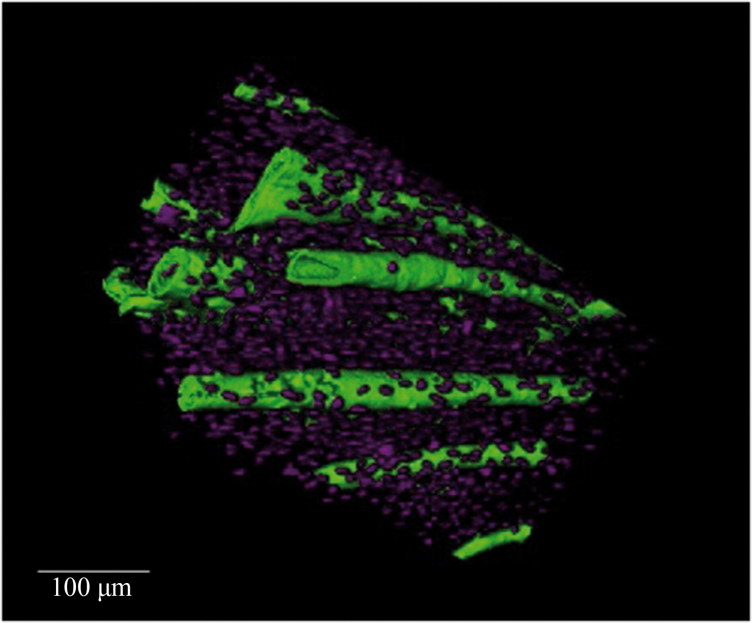
Synchrotron radiation-based micro-CT 3D render of a cortical bone Volume of Interest from a human mandible. Vascular canals (green) and osteocyte lacunae (purple) are visualized. Scale = 100 μm. Credit: JM Andronowski. Reprint permission granted by the publisher. (For interpretation of the references to colour in this figure legend, the reader is referred to the web version of the article).

**Figure 3.**
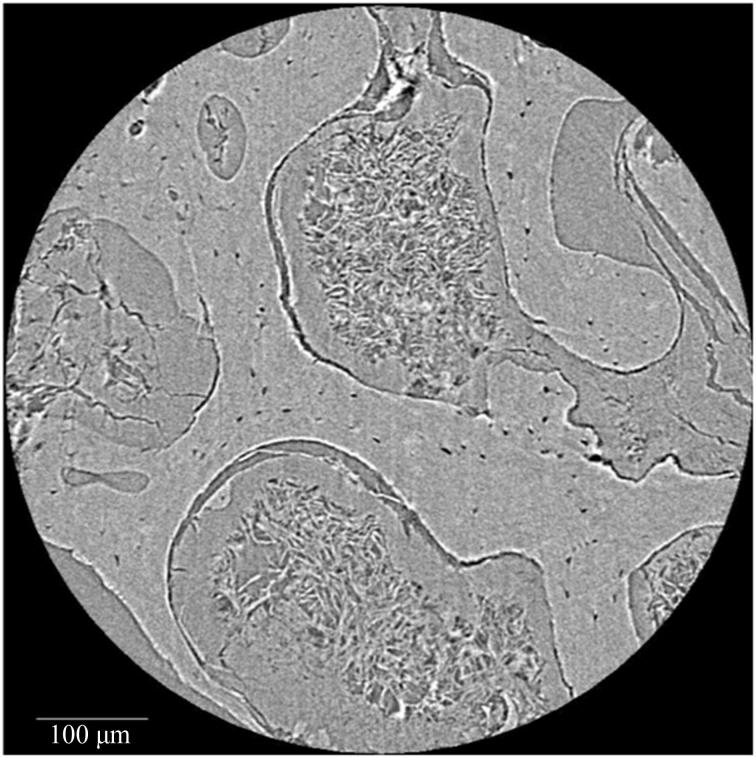
Synchrotron radiation-based micro-CT single projection of a cancellous bone cylindrical Volume of Interest. Probable soft tissue remnants are evident between the trabecular struts. Scale = 100 μm. Credit: JM Andronowski. Reprint permission granted by the publisher.

Research by Andronowski et al. [72] investigated whether differences in 3D bone microstructure may be used to explain differential nuclear DNA yield among bone tissue types (cortical and cancellous bone), with a focus on osteocytes and the 3D quantification of their associated lacunar spaces. Osteocytes and other bone cells are recognized to house nuclear DNA in bone tissue, thus examining the density of their lacunae was explored to potentially explain why DNA yield rates differ among bone tissue types. Identifying which bone tissue type(s) and/or bone envelope(s) (e.g. periosteal, intracortical, and endosteal) provide the highest nuclear DNA yields will further inform current bone-sampling protocols for human identification and limit the amount of bone tissue necessary for DNA analysis. Results demonstrated that osteocyte lacunar abundance and density vary between cortical and cancellous bone tissue types, with cortical bone VOIs containing a higher lacunar abundance and density (Figure 2). The osteocyte lacunar density values are independent of nuclear DNA yield, suggesting an alternative explanation for the higher nuclear DNA yields from bones with high quantities of cancellous bone. A plausible explanation focuses on remnants of soft tissue between trabeculae observed using SR micro-CT. Although soft tissue was not present on the surface of the bones, 3D scans consistently revealed probable soft tissues within the medullary cavities of skeletal elements with high cancellous content (Figure 3). It is hypothesized that these residual soft tissues, which likely include endosteum and osteological lining cells, contributed to the higher nuclear DNA yields from cancellous bone.

**Figure 4.**
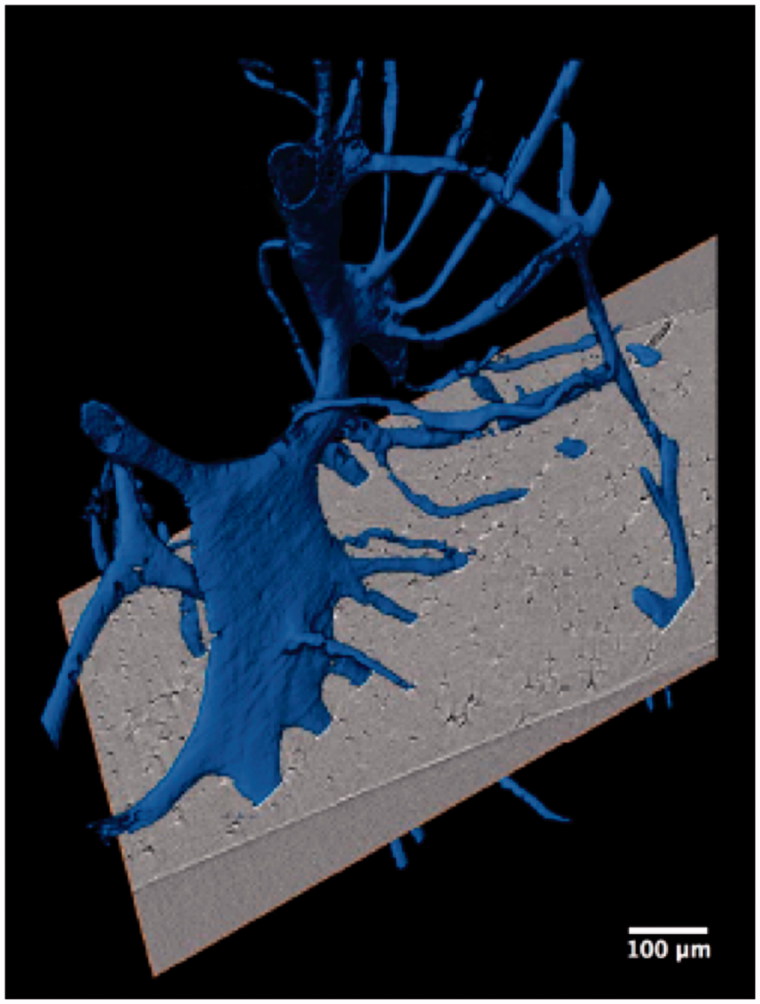
Synchrotron radiation-based micro-CT 3D render of adjacent interconnecting osteonal canal networks (blue). A 2D slice is included to demonstrate their contributions to a complex osteon band. Scale = 100 μm. Credit: JM Andronowski. Reprint permission granted by the publisher. (For interpretation of the references to colour in this figure legend, the reader is referred to the web version of the article).

**Figure 5.**
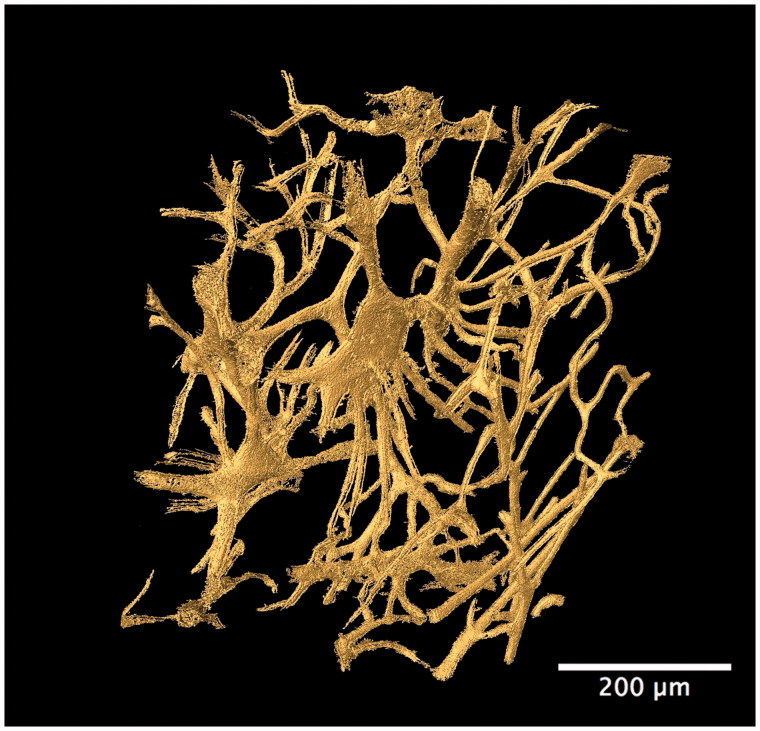
Synchrotron radiation-based micro-CT 3D render of the cortical bone vascular canal network from a human temporal bone specimen (gold). A high degree of interconnectivity among the entire vascular network is evident. Scale = 200 μm. Credit: JM Andronowski. Reprint permission granted by the publisher. (For interpretation of the references to colour in this figure legend, the reader is referred to the web version of the article).

Other studies in forensic anthropology have focused on the evaluation of bone organization as a means to differentiate species. For example, Mulhern and Ubelaker [80] identified osteon banding as a feature that can differentiate human from non human bone. Multiple rows of lengthy primary or secondary osteon chains have been described as diagnostic of non human bone, particularly the occurrence of multiple bands. Additional research by Andronowski et al. [73] using SR micro-CT, however, documented the presence of osteon banding in cortical bone from adult males. Linear arrangements of primary and/or secondary osteons and the presence of multiple bands were observed in various bone types. A particularly interesting finding was that osteonal canal networks were seen to interact with adjacent networks, contributing to a single osteon band. A 3D render of two such interconnecting osteonal canal networks can be visualized in Figure 4, demonstrating their complexity. High degrees of interconnectivity among entire vascular canal networks (Figure 5) were observed owing to the continuous process of bone remodelling and branching. Although osteon banding can be suggestive of non human bone, the frequent occurrence of osteon banding and the presence of multiple bands within single human specimens in this study, indicate that osteon banding alone is not diagnostic of non human bone.

The 3D examination of bone microarchitecture also holds promise for examining bone loss in past populations. Scholars have warned against the use of certain clinical technologies, particularly dual energy X-ray absorptiometry (DXA), for the examination of bone loss in archaeological remains. In comparison with many other histological techniques such as light microscopy, DXA performed poorly [60]. As such, using 3D imaging techniques provide a more accurate and direct comparison between archaeological and modern samples.

The application of non-destructive 3D imaging technologies to biomedical and bone biology research is considerable and offers tremendous future potential to the field of anthropology. There are specific advantages of synchrotron-based techniques that have further created the potential to image cortical porosity *in vivo* and over time, offering an avenue into the fourth dimension.

## Future perspectives: *in vivo* imaging

### High-resolution peripheral quantitative computed tomography

A unique opportunity to explore human bone tissue *in vivo* has been provided by a new generation of clinical scanners. High-Resolution Peripheral Quantitative Computed Tomography (HR-pQCT) systems have been introduced in clinical settings to assess the integrity of cancellous bone microstructure for osteoporosis research. More recent technological developments have led to HR-pQCT systems with much higher spatial resolutions and *in vivo* imaging capabilities of human cortical cross-sectional properties [81, 82]. For example, Boutroy et al. [81] assessed bone density and quantified bone microarchitectural measurements at peripheral skeletal sites (e.g. distal radius and tibia). Their study sample included healthy premenopausal women, and osteopenic and osteoporotic postmenopausal women, and employed *in vivo* cross-sectional longitudinal scans. While HR-pQCT has been significant in clinical studies focused on osteoporosis-related change in cancellous bone and specific cortical microstructural parameters, it does not possess the ability to track remodelling events over time [83]. Owing to resolution limitations, frequent restriction to cancellous bone regions, and artifacts associated with movement of the subject during scanning, longitudinally assessing BMUs in cortical bone is not currently possible [37].

### Micro-CT: in vivo

First implemented in the 1990s, *in vivo* micro-CT employed high-resolution imaging of small animal cancellous bone using monochromatic X-ray sources. This approach allows for the quantification of 3D bone microstructure *in vivo*. As such, early studies lead to the development of commercial desktop *in vivo* micro-CT laboratory scanners [84]. This technique is a greatly advantageous as it affords the ability to track changes in bone mass and microarchitecture over time, and subjects can act as their own controls [85]. Using this imaging modality, projects have been developed that allow for the implementation of longitudinal studies. This is a tremendous improvement over traditional 2D cross-sectional methods, which can be clouded by variability within subject groups [85]. A longitudinal approach permits researchers to examine direct observations of a particular development in single animals, and bone formation and resorption can be quantified across the bone matrix. Synchrotron X-ray sources can also be employed to *in vivo* micro-CT. The earliest studies employing this technique were completed in the mid-1990s by a research group at the Stanford Synchrotron Radiation Laboratory. A proof-of-principle study from the University of Saskatchewan explored the spatio-temporal regulation of bone remodelling using *in vivo* micro-CT through the employment of a fatigue-loading model in laboratory animals [37].

A primary limitation of *in vivo* micro-CT using live subjects, however, is the issue of radiation dose on study animals. Generally speaking, the radiation dose is a measure of how much energy is left behind in the tissue by the X-ray beam. Absorbed dose, however, is the energy deposited per tissue mass and this is measured in Grays (Gy). To increase understanding of repeated radiation exposure on bone loss, Klinck et al. [85] examined the radiation effect in various mice and rat species. The goal of this work was to provide information for researchers designing future *in vivo* studies involving repeated radiation exposure. The authors explained that the absorbed radiation dose is proportional to the degree of resolution, and any increase in resolution will involve an increased dose of radiation. For this reason, researchers have been unable to image cortical bone microarchitecture *in vivo* since higher resolutions are needed to clearly image the cortical pore network [36]. This, and comparable work, are essential to consider when imaging live animals. Additionally, working with live animals presents challenges in terms of stage set-up and security of the device used to hold the animal still. Animals must be imaged under anaesthetic to avoid movement that may introduce artifacts in the reconstructed images.

Tracking bone remodelling has the potential to be transformative for our understanding of bone physiology and disease [37]. A novel opportunity exists to develop new approaches to histological aging which would benefit from considering the dynamic nature of bone and evaluating the spatial relationship of osteons within cortical bone.

## Future perspectives: exploring bone quality using non-histological modalities

By taking a holistic approach to understanding bone microstructure, research is expanding to alternative modalities that explore the extrinsic and intrinsic properties of bone. Coupling alternative techniques with bone histology and 3D imaging provides a more intricate picture of bone biology. From 2013 to present, there have been a series of research projects performed at the Harris County Institute of Forensic Sciences by forensic anthropologists Drs Christian Crowder, Jennifer Love, and Miriam Soto Martinez focusing on exploring paediatric bone quality to assist with the evaluation of accidental and non-accidental skeletal injury. While these studies do not address histological structure, they focus on understanding bone distribution and composition, and their relationship to fracture resistance in paediatric bone. Below is a description of various modalities used in these studies. Publications from these projects are currently underway, thus results from the studies are not provided.

### Techniques for evaluating bone quality

Bone quality has been described as the “totality of features and characteristics that influence a bone’s ability to resist fractures” [86]. Bone quality encompasses several characteristics of bone, such as bone tissue quantity, the spatial distribution of bone mass (size, shape, architecture), and material composition. There is no single technique that can provide a comprehensive and quantifiable measurement of bone quality due to the numerous factors that contribute to it. As a result, the measurements of the individual characteristics of bone are used as surrogate measures of bone quality. The most common surrogate measure is BMD, a measure of bone quantity [67]. Although important, BMD only explains a portion of overall bone quality. The prediction of bone strength and fracture risk is improved when measures of bone architecture and composition are included [87, 88]. Bone architecture at the macroscopic and microscopic level describes the spatial distribution of bone mass. At the macroscopic level, the distribution of bone mass is quantified using geometric measurements, such as cross-sectional measurements. Measurements, such as cortical porosity and trabecular number, capture the spatial distribution of bone mass at the microscopic level. Material composition and the arrangement of these molecules also significantly influence bone quality considering that a structure is only as strong as the materials used to construct it. The bone material composition is evaluated by measuring factors such as bone mineral to collagen ratio, carbonate to phosphate ratio, and mineral crystallinity. The integration of these factors is responsible for the structural and material properties of bone, and hence the ability to resist fracture [89]. The influence of bone characteristics on fracture resistance can be evaluated by comparing measurements of these characteristics to the structural (extrinsic) and material (intrinsic) properties of bone calculated by mechanical testing, such as bone tissue stiffness (elastic modulus) and the maximum loading that bone tissue can sustain without fracturing (ultimate stress) [90]. Techniques used to measure the various characteristics of bone quality and the material and structural properties of bone include DXA, CT (QCT, HR-pQCT, micro-CT), as described above, Raman spectroscopy, and quantitative ultrasound (QUS).

## Dual energy X-ray absorptiometry

DXA uses two small doses of ionizing radiation (low energy X-rays) to measure the density of tissue. The X-rays are absorbed differently by the soft tissues and hard tissues (bones). DXA measures BMD with a high level of accuracy and precision in adults. Thus, well-established DXA reference data are available for adults; however, paediatric DXA scans are more difficult to interpret. DXA is an areal (2D) rather than a volumetric density measure and the non-uniform growth of bones can introduce error in BMD measurements. Also, the prognostic value of paediatric DXA with regards to fracture risk or peak BMD has not been established [91]. For clinicians, developing DXA to measure bone mineral density and content in children is extremely important considering that it is minimally invasive and provides little exposure to radiation. In forensic anthropology, this modality has the potential to assist with providing evidence of child abuse (malnourishment) and understanding fracture risk in paediatric skeletal trauma cases.

### Raman spectroscopy

Raman spectroscopy functions by focusing the incident laser light on a bone specimen causing the chemical bonds in the mineral and protein components of the bone material to vibrate. The vibrations of the molecules cause a small fraction of light to lose energy at which point it is scattered at a longer wavelength [92]. The difference in wavelength between the incident light and the scattered light corresponds to molecular vibrations and lead to characteristic frequency shifts in the Raman spectrum. These frequency shifts are recorded as absorption peaks on a Raman spectrum, which characterize the material composition of the tissue sample. The high resolution and ability to analyze re-hydrated tissues are advantages of this technique.

**Figure 6.**
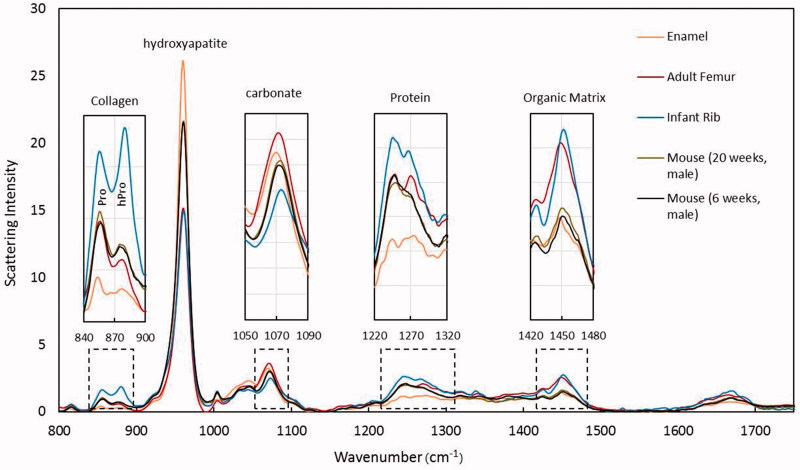
Examples of Raman spectroscopy wavelength graph comparing the following samples: enamel, human adult femur, human infant rib, mouse bone 20 weeks, and mouse bone 6 weeks. From left to right the graphs shows mineral-to-collagen ratio (Proline and Amide III), mineral crystallinity of the hydroxyapatite, carbonation, collagen content (protein), and collagen cross-linking (organic matrix). Credit: Dr Bi Xiaohong, with permission.

Raman spectroscopy measures components of bone tissue that are key determinants in bone quality and strength [93], such as the mineral to matrix ratio, carbonate to phosphate ratio, collagen crosslink ratio, and mineral crystallinity [67] (Figure 6 and Table 1). The compositional measurements obtained by this modality are correlated with cortical bone stiffness, bending strength, and yield stress [94]. The mineral to matrix ratio indicates the degree of tissue mineralization. The carbonate to phosphate ratio provides a measure of the substitution of carbonate ions in phosphate positions within the hydroxyapatite molecules. A higher degree of carbonate substitution has been found in individuals with osteoporosis than in individuals with normal bone [95], suggesting that carbonate substitution may weaken bone tissue. The ratio of non-reducible to reducible bonds (crosslinks) between collagen molecules within bone is a measure of collagen quality. Collagen crosslinks are important because they are responsible for bone’s strength in tension. Bone samples from premenopausal women with fragility fractures, but normal BMD, have been shown to have greater non-reducible to reducible collagen crosslink ratios than bone samples from normal male and female adults (51–70 years of age) [96]. During mineralization, mineral crystals form a lattice network. The size and distribution of mineral crystals within the lattice network may affect susceptibility to fracture by inducing local strains on adjacent mineral crystal and collagen molecules. Increased mineral size is correlated with decreased tissue strength of aging cortical bone [94].

**Table 1.** A description of molecular composition and bone properties for mineral-to-collagen ratio (proline and amide III), mineral crystallinity of the hydroxyapatite, carbonation, collagen content (protein), and collagen cross-linking.

Bone parameters	Molecular composition and bone properties
Mineral-to-collagen ratio (Pv1/Proline, Pv2/AmideIII)	Relative amount of bone mineral to organic matrix; typically positively correlated with bone strength; lower values for new bones
Mineral crystallinity	Perfection or size of mineral crystals: typically positively correlated with bone strength; lower values for new bones
Carbonation	Substitution of carbonate ions in phosphate positions of hydroxyapatite; an indicator of mineral maturation; higher values for aged bones
Collagen content	Relative amounts of collagen to organic matrix; higher values for new bones
Collagen cross-linking	Relative amounts of collagen cross-linking to organic matrix

## Quantitative ultrasound

QUS may offer a refined alternative to other techniques as a quantitative method for evaluating infant bone quality and fracture risk [97–109]. QUS devices measure the speed of sound (SOS, m/s) of an ultrasound wave as it is transmitted through bone or along the bone surface. Some QUS devices also measure the broadband attenuation (BUA, dB/MHz) of the signal strength as it travels through the bone. Several studies suggest that SOS is directly related to bone quality as it is influenced by material and structural properties that determine bone strength [98–101, 110]. Specifically, BMD, bone stiffness (elastic modulus), cortical thickness, micro-architecture, and fatigue damage are significantly correlated with SOS values [111–117]. Research further indicates that SOS is correlated with bone strength measured through biomechanical testing [118–120].

In adults, QUS is currently employed as a screening tool for osteoporosis [121–126]. Clinical studies demonstrate that SOS is capable of discriminating healthy individuals from those with diseased bone [120, 127–130]. Furthermore, SOS measurements are capable of predicting fracture risk independently of BMD [122, 131], indicating that SOS is affected by aspects of fracture resistance that are not captured by BMD alone.

QUS is not currently applied in the clinical setting to assess bone quality and fracture risk in infants. Standard protocols and normal bone thresholds have not been developed for infants and young children, though research indicates that it may be a promising tool for the evaluation of fracture resistance in the paediatric population [98–109]. QUS performed as well as DXA in identifying low BMD in children with fragility fractures [132] and better than bone mineral content at predicting fractures in isolated infant bones *in vitro* [133]. While previous research suggests that QUS may offer an alternative avenue for evaluating paediatric fracture resistance, much of the SOS literature on infant bone focuses on term infants during the immediate neonatal period and preterm infants at birth and term-corrected age [97–100, 102, 103, 105–107, 133–139]. Longitudinal studies are also: (1) limited by small sample sizes, (2) restricted to preterm or very low birth weight infants, or (3) provide data from SOS measurements obtained at inconsistent measurement intervals [101, 104, 106, 108, 109, 140–144].

Overall, the relationship between paediatric bone biology and SOS needs further evaluation, especially in infants. In the paediatric population, research relating SOS to bone quality indicators, such as material composition, cross-sectional measurements, and direct measurements of bone strength is lacking. While normative reference databases are available for QUS devices, the number of individuals in each age cohort, by age in months, is unknown. Published information on these reference databases is only specific to age in years. Studies that have examined SOS in infants show that SOS changes significantly over the first year of life [144, 145]. As such, it is important to develop SOS ranges specific to age in months. Further research is needed to evaluate whether QUS effectively measures fracture resistance. SOS needs to be compared with other, more direct, measures of bone quality indicators such as mechanical properties of infant bone.

## Conclusion

Bone is a mechanically active, three-dimensionally complex, and dynamic tissue that changes in structure over the human lifespan. The remodelling process is carried out by BMUs and is the primary means of skeletal adaptation in adults. Until recently, the evaluation of bone microarchitecture at the micron-scale and analysis of cortical bone remodelling has been focused within the 2D realm. There is a great strength in applying a holistic approach to understanding bone microstructure by combining 2D histological and 3D imaging approaches, and including alternative modalities that explore the extrinsic and intrinsic properties of bone. Integrating techniques with bone histology provide a more intricate picture of bone biology and maximizes the information available. In employing this combined approach, the best of each modality will enable targeted histology to be directed by 3D imaging. Insights revealed by 3D data will allow the bone biology community to shed new light on the processes of bone aging and disease, and help to inform the development of future histologic age estimation methods in the field of forensic anthropology.
